# When is it considered reasonable to start a risky and uncomfortable treatment in critically ill patients? A random sample online questionnaire study

**DOI:** 10.1186/s12910-021-00705-4

**Published:** 2021-11-03

**Authors:** M. Zink, A. Horvath, V. Stadlbauer

**Affiliations:** 1 Department of Anaesthesiology and Intensive Care Medicine, Hospital of the Brothers of St. John of God, St. Veit an Der Glan, Austria and Hospital of the Elisabethinen Klagenfurt, Klagenfurt, Austria; 2 Department of Internal Medicine, Research Unit “Transplantation Research”, Medical University of Graz, Graz, Austria; 3 Center for Biomarker Research in Medicine (CBmed), Graz, Austria; 4 Department of Internal Medicine, Division of Gastroenterology and Hepatology, Auenbruggerplatz 15, 8036 Graz, Austria

**Keywords:** Inappropriate treatment, Futility, Critical care, Decision making, Sex, Demographic, Health care professional

## Abstract

**Background:**

Health care professionals have to judge the appropriateness of treatment in critical care on a daily basis. There is general consensus that critical care interventions should not be performed when they are inappropriate. It is not yet clear which chances of survival are considered necessary or which risk for serious disabilities is acceptable in quantitative terms for different stakeholders to start intensive care treatment.

**Methods:**

We performed an anonymous online survey in a random sample of 1,052 participants recruited via email invitation and social media. Age, sex, nationality, education, professional involvement in health care, critical care medicine and treatment decisions in critical care medicine as well as personal experience with critical illness were assessed as potential influencing variables. Participants provided their opinion on the necessary chances of survival and the acceptable risk for serious disabilities to start a high-risk or uncomfortable therapy for themselves, relatives or for their patients on a scale of 0–100%.

**Results:**

Answers ranged from 0 to 100% for all questions. A three-peak pattern with different distributions of the peaks was observed. Sex, education, being a health care professional, being involved in treatment decisions and religiosity influence these opinions. Male respondents and those with a university education would agree that a risky and uncomfortable treatment should be started even with a low chance of survival for themselves, relatives and patients. More respondents would choose a lower necessary chance of survival (0–33% survival) when deciding for patients compared to themselves or relatives to start a risky and uncomfortable treatment. On the other hand, the majority of respondents would accept only a low risk of severe disability for both themselves and their patients.

**Conclusion:**

No cut-off can be identified for the necessary chances of survival or the acceptable risk of disability to help quantify the “inappropriateness” of critical care treatment. Sex and education are the strongest influencing factors on this opinion. The large variation in personal opinions, depending on demographic and personality variables and education needs to be considered in the communication between health care professionals and patients or surrogates.

**Supplementary Information:**

The online version contains supplementary material available at 10.1186/s12910-021-00705-4.

## Background

In medicine, especially in critical care medicine, fundamental decisions about the therapy strategy have to be made by health care professionals on a daily basis. The concept of inappropriateness of treatment, often also called “futility”, arises once it is realised that therapeutic measures may be technically feasible in critical care medicine but may not benefit a patient [1]. Decisions on the appropriateness of intensive care treatment should be based on fundamental ethical principles such as respect for the autonomy and dignity of the patient [2–4]. Interventions should aim for the well-being of the patient with avoidance of harm as the highest priority as well as fair use of available means [5]. Decisions on the appropriateness of therapies are therefore a common practice in critical care medicine and recommended by practice guidelines [6]. Generally, critical care interventions should be considered inappropriate when there is no reasonable expectation that the patient will improve sufficiently to survive outside the acute care setting or when there is no reasonable expectation that the patient’s neurologic function will improve sufficiently to allow the patient to perceive the benefits of treatment [7]. While the theoretical concept of “futility” or “inappropriate treatment” [8] to guide physicians as to when they should or even must cease aggressive treatment is generally accepted, the practical definition is difficult and may lead to uncertainty [9–11]. To overcome uncertainties, such decisions are made after weighing up all risks and benefits. Evidence-based and shared decision making are common practice in critical care [12, 13]. The implementation of state laws, recommendations from professional associations or hospital policies that provide a transparent and fair process to considering ethical and medical aspects can help settle such disputes. The Austrian consensus recommendations on therapy limitation and therapy discontinuation in intensive care units, as an example, state that a planned admission to ICU is to be based on the existence of a positive prospect of patient survival [1]. This requires the accurate prediction of outcome after the intervention, which is not possible in many cases [14]. The use of prognosis scores alone is also not considered ethically appropriate because the scores are not developed for the purpose of deciding on “inappropriateness of therapy” and do not consider the complete picture of an individual patient [15]. Besides the medical uncertainty in assessing prognosis accurately, there may also be controversy on the appropriateness of treatment between health care professionals and patients or, more often, surrogates, as critically ill patients often can no longer communicate their wishes [8, 16]. Furthermore, prediction of “appropriate” positive prospect of a treatment also contains a personal component and may have high individual variability in the interpretation of an acceptable outcome regarding quality of life. It is not exactly known which personal and professional factors influence the perception of “appropriateness” of treatment in critical care.

Therefore, we conducted an online survey on a random sample of participants to learn which chances of survival are considered necessary and which risk for serious disabilities is acceptable in quantitative terms for different stakeholders to start intensive care treatment.

## Methods

First, a literature search was performed in PubMed and PubPsych to identify a validated questionnaire. Because no appropriate validated questionnaire could be identified from literature, a web-based questionnaire in German and English was developed by two of the authors (MZ and VS) (see Additional file 1). Face validity, feasibility and utility were tested with 10 people (5 medical and 5 non-medical professionals unrelated to the study) before fielding the questionnaire. Translation into English was performed by VS, and then the questionnaire was proofread by a native speaker and translated back to German by MZ to ensure correct translation. The questionnaire consisted of 11–13 questions. Of these, eight questions related to demographic characteristics and professional and personal exposure to critical care treatment. Another 3–5 questions then assessed the individual survival chances and risk of severe disability the respondents would accept when undergoing a risky or uncomfortable treatment. Participants were asked to imagine that they are in the hospital with a life-threatening disease and to give their opinion for which chances of survival they would undergo a risky and uncomfortable treatment on a slide scale between 0 and 100%. Next, they were asked to choose for a close friend or relative. Finally, all participants were asked to imagine that they are in a situation for which a risky and uncomfortable treatment will ensure survival, but there is a risk of severe disability after the treatment. They were asked which risk of severe disability with the need for long-term care they would consider acceptable. Those who stated that they were health care professionals and professionally involved in treatment decisions of critically ill patients were also asked to imagine that they are treating a critically ill patient who is unconscious and to decide which chances of survival they would consider necessary to begin a risky and uncomfortable treatment on this patient and which risk of severe disability with the need for long-term care they would consider acceptable.

The questionnaire was distributed via email to different organisations, clubs and religious communities by MZ (for detailed information see Additional file 1) and it was posted on Facebook by VS on her profile as well as in a Austrian Facebook group for physicians (Ärzte vs Covid-19) between 16.9.2016 and 11.07.2020. In all mail and postings, recipients were also asked to actively share the information and invite others to participate in the survey. The chosen method of distribution does not allow us to determine the number of people who received the invitation to participate in the survey. We aimed for 1,000 complete answers and assumed that 20% of the surveys will be incomplete. Sample size was chosen based on feasibility. Therefore, the survey was closed after approximately 1,200 surveys were returned. The duration of data collection was considerably longer than initially expected because the response rates from the email invitations were lower than expected. SurveyGizmo (Boulder, CO 80301 USA, now renamed to Alchemer) was used for data collection. The study was approved by the research ethics committee of the Medical University of Graz (28-462 ex 15/16). Data collection was performed anonymously without logging any personal identifiers. SPSS V26 (IBM, Armonk, NY, USA) was used for analysis. Visualization was performed in R V3.5.2 software (“ggplot” package) in Rstudio 1.3.1056 [17, 18]. Data are presented as absolute numbers, percentages or median with 95% confidence interval. Univariate inter-group data analysis using Chi-square test was used for categorical data. Normality testing was performed using Shapiro–Wilk test for continuous data. Because the variables were not normally distributed, Mann–Whitney U test was used to compare groups. In addition, multinomial logistic regression was performed to identify which factors could predict the chances of survival with and without sever disability that were considered appropriate to undergo risky treatment. Nationality was grouped as “Austria” or “other” for the analysis, and education was grouped into university or other (combining high school, apprenticeship and compulsory education) due to the sample size. A *p* < 0.05 was considered statistically significant.

## Results

### Characteristics of survey participants

A total of 1,223 people started the questionnaire, 164 only clicked on the link of the questionnaire and 7 started to answer the questions but did not submit; hence, 1,052 questionnaires were available for analysis. The mean age of respondents was 44 (44;45), 396 (37.6%) were male, 653 (62%) were female and 3 (0.3%) preferred not to tell. Male respondents were significantly older than female (46 (44;49) versus 42 (41;44) years, *p* < 0.0001). In this sample, 531 (51%) of the respondents considered themselves as religious, 510 (49%) said that they are not religious and 11 (1%) did not answer this question. The majority (693; 66%) reported a university degree as highest education followed by apprenticeship (202; 19%) and high school education (140; 13%). By far, most respondents were from Austria (932; 89%) while a few were from Germany (38; 4%), Italy (14; 1%) and Switzerland (13; 1%), and the remaining participants were from 24 countries with no more than 6 respondents from the same country. More women than men (54% versus 47%, *p* = 0.025) reported that they are religious, more male respondents came from countries other than Austria (16% versus 7%, *p* < 0.0001) and more men had a university education (77% versus 60% of the women, *p* < 0.0001). Table 1 summarizes the characteristics of the participants.

**Table 1:** Description of the study population

	N	Percentage (from total participants n = 1052)
Sex		
Female	653	62.1
Male	396	37.6
No answer	3	0.3
Religion		
Christian	890	84.6
Atheist	108	10.3
Buddhism	10	1.0
Islam	2	0.2
Other	30	2.9
No answer	12	1.1
Religious		
Yes	531	50.5
No	510	48.5
No answer	11	1
Country		
Austria	932	88.6
Germany	38	3.6
Italy	14	1.3
Switzerland	13	1.2
Other^a^	43	4.1
No answer	12	1.1
Education		
University	693	65.9
High school	140	13.3
Apprenticeship	202	19.2
Compulsory education	14	1.3
No answer	3	0.3

^a^Algeria, Argentina, Australia, Azerbaijan, Bahamas, Bosnia and Herzegovina, Bulgaria, Burkina Faso, Canada, Croatia, Czech Republic, Greece, Hungary, India, Kazakhstan, Netherlands, Poland, Romania, Serbia, Slovakia, Slovenia, South Korea, Spain, Thailand

**Fig. 1:**
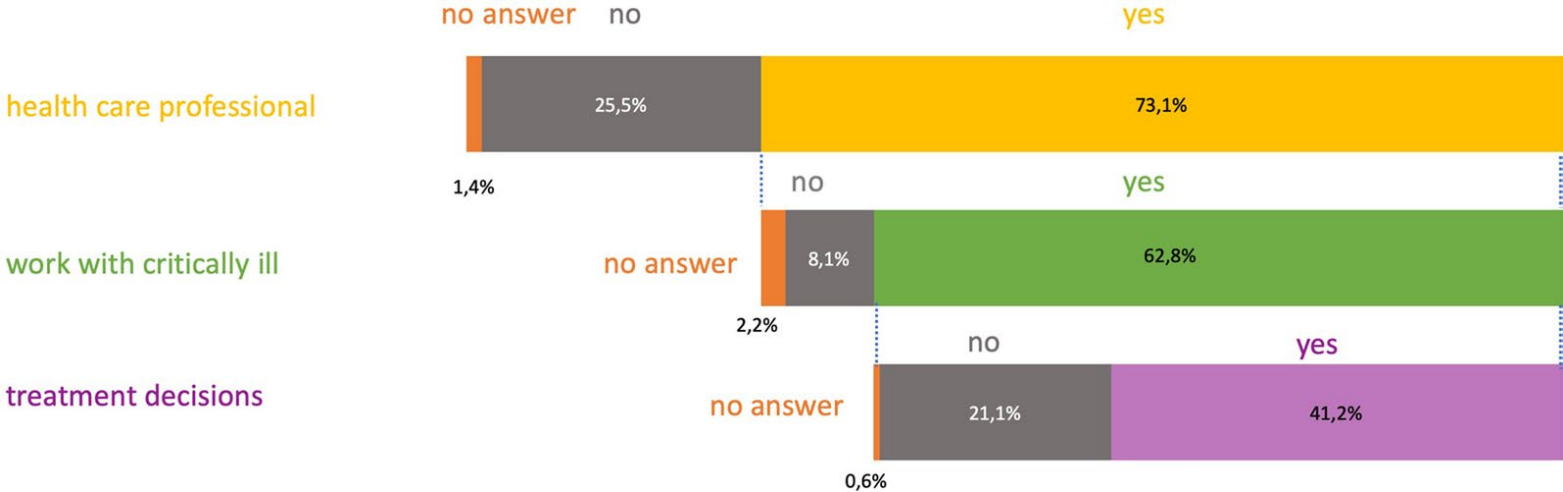
Distribution of answers in relation to respondents’ professional involvement with critically ill patients

The majority of the respondents were health care professionals (73%) and from those the majority (86%) reported to work with critically ill patients. From those who reported to work with critically ill patients, 66% reported that they are professionally involved in treatment decisions in critically ill patients (Fig. 1). For all respondents, 22% reported that they already were critically ill or in a life-threatening situation themselves and of those, 66% were personally involved in their treatment decisions. For all respondents, 78% reported that a closely related person has been critically ill or in a life-threatening situation and of those, 59% stated that they were personally involved in treatment decisions. Male respondents were more likely to work with critically ill patients than women (92% versus 86%, *p* = 0.017) and to be professionally involved in treatment decisions (84% versus 56%, *p* < 0.0001). More people who reported that they had been critically ill themselves also considered themselves to be religious (60% versus 48% who were not critically ill, *p* = 0.002). A similar association was found for those who reported that one of their relatives was critically ill (53% versus 44% who had no relatives who were critically ill, *p* = 0.002). Level of education was significantly associated with working as a health care professional (university 82%, high school 45%, apprenticeship 69%, compulsory education 50%, *p* < 0.0001), working with critically ill (university 91%, high school 76%, apprenticeship 86%, compulsory education 57%, *p* < 0.0001) and being professionally involved in treatment decisions (university 83%, high school 20%, apprenticeship 13%, compulsory education 25%, *p* < 0.0001).

### Necessary survival chances and acceptable risks of intensive care treatments

**Fig. 2:**
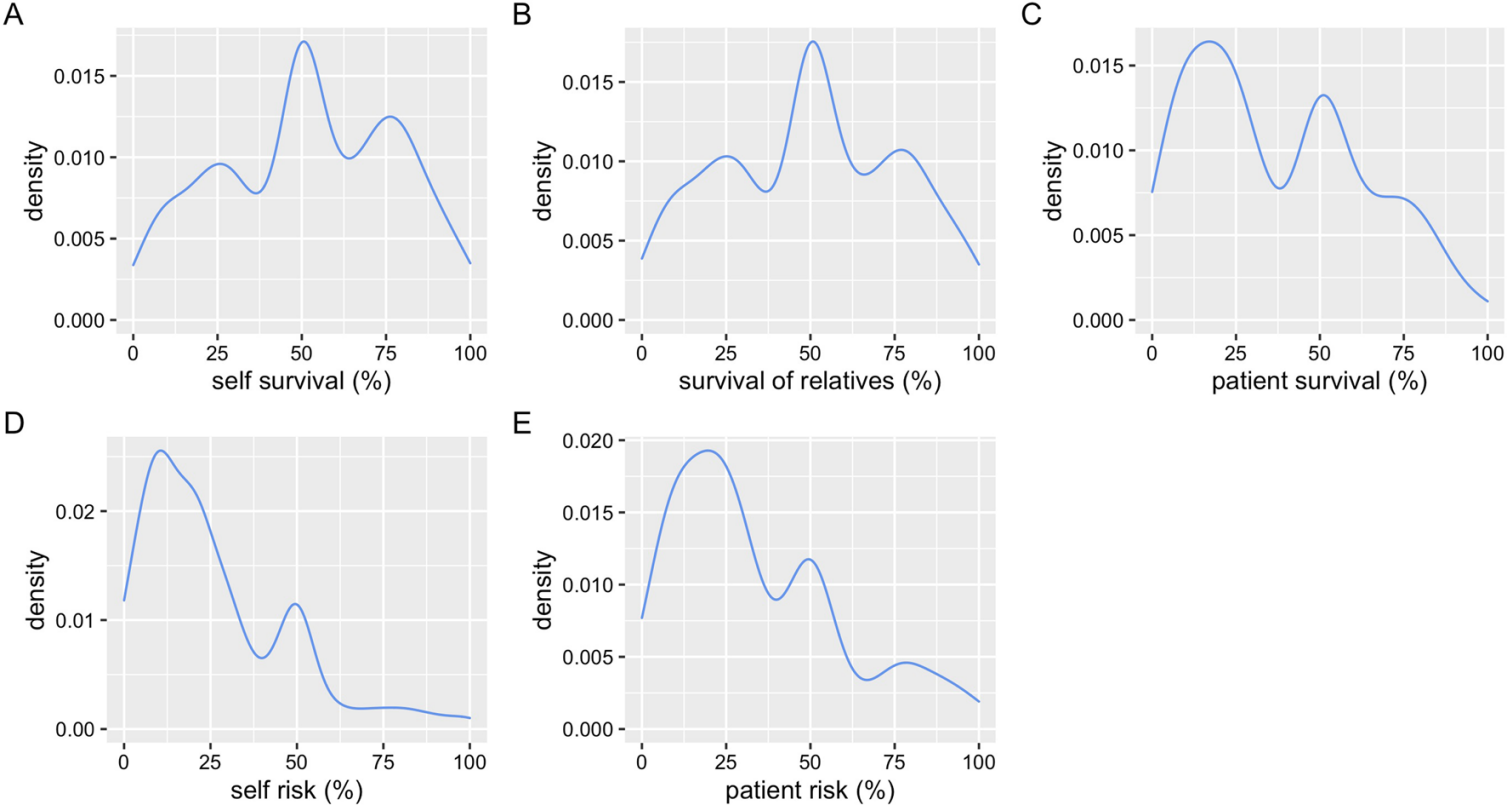
Density plots showing the distribution of responses to the different questions: **A** necessary survival for the respondents themselves to undergo a risky or uncomfortable therapy when critically ill; **B** necessary survival for close relatives to undergo a risky or uncomfortable therapy when critically ill; **C** necessary survival for critically ill patients to undergo a risky or uncomfortable therapy; **D** acceptable risk for severe disability for respondents themselves; **E** acceptable risk for severe disability for patients

Responses regarding necessary chances of survival and acceptable risks of intensive care treatments covered the complete span from 0 to 100%. The distribution shows a triphasic pattern for all answers in the density plots (Fig. 2A–E). Responses regarding the necessary chances of survival for the respondents themselves and for their relatives showed the highest density in the middle tercile while the answers regarding necessary survival of patients and acceptable risk for disability for themselves and patients showed the highest peak in the lowest tercile (Table 2).

**Table 2:** Distribution of answers regarding necessary survival chances and acceptable risks of critical care treatments (grouped into terciles; low 0–33%, medium 34–66%, high 67–100%)

	N	Percentage (%)
Self survival		
Low	298	29.0
medium	383	37.2
high	348	33.8
Relative survival		
Low	332	32.1
Medium	390	37.7
High	312	30.2
Patient survival		
Low	222	52.5
Medium	128	30.3
High	73	17.3
Patient risk		
Low	251	61.8
Medium	99	24.4
High	56	13.8
Self risk		
Low	676	73.0
Medium	195	21.1
High	55	5.9

### Influence of demographic factors on responses regarding necessary chances of survival and acceptable risks of critical care treatments

**Fig. 3:**
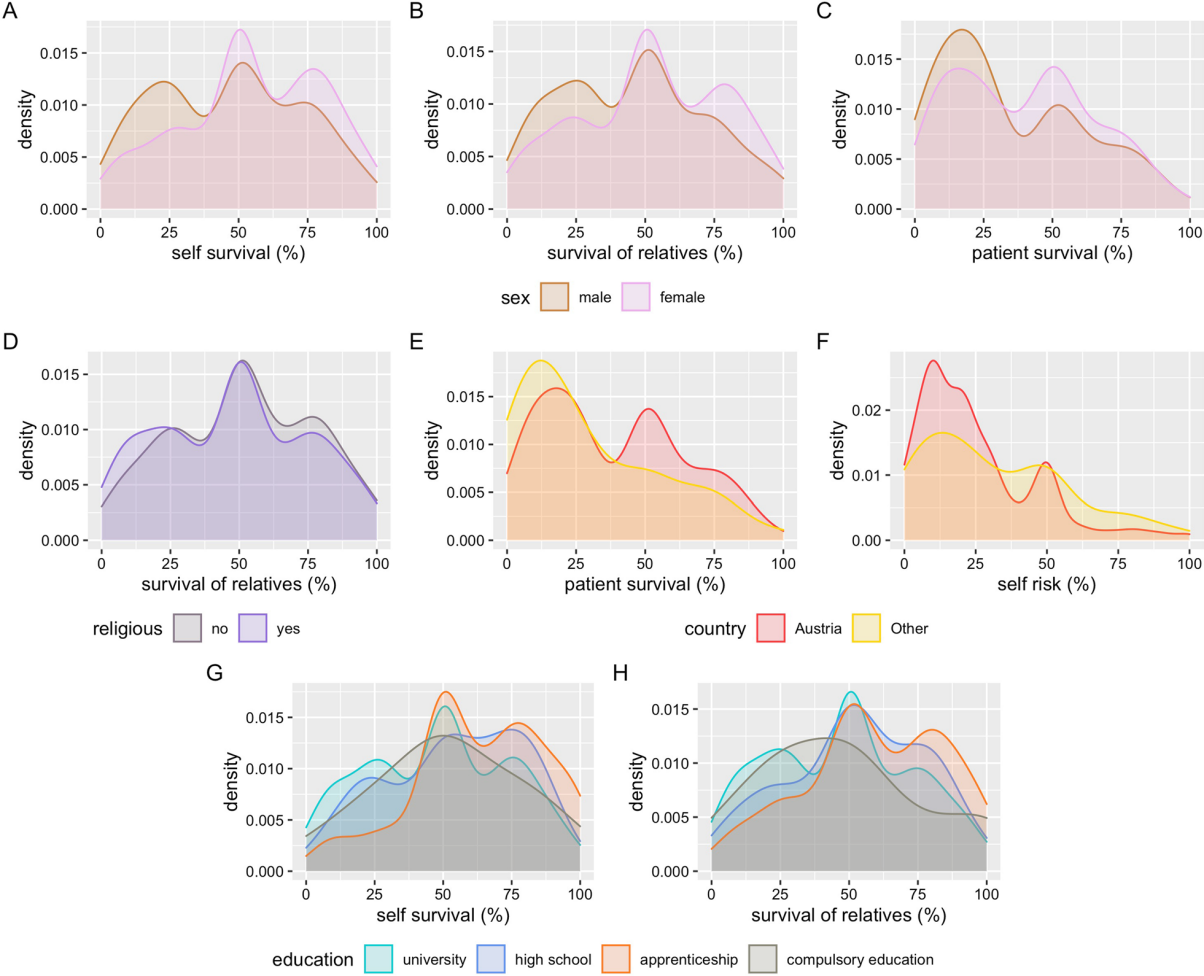
Density plots showing the distribution of responses to the different questions in relation to different demographic variables (only significant differences are shown): **A**–**C** necessary survival for the respondents themselves, relatives and patients to undergo a risky or uncomfortable therapy when critically ill in relation to sex of the respondents; **D** necessary survival for relatives to undergo a risky or uncomfortable therapy when critically ill in relation to being religious or not; **E**, **F** necessary survival for patients to undergo a risky or uncomfortable therapy when critically ill and acceptable risk for severe disability for respondents themselves in relation to country of origin of the respondents; **G**, **H** necessary survival for the respondents themselves and for relatives to undergo a risky or uncomfortable therapy when critically ill in relation to education of the respondents

Male respondents would accept a lower chance of survival necessary to undertake a risky and uncomfortable treatment for themselves (*p* < 0.0001), for their relatives (*p* < 0.0001) and for patients (*p* < 0.039) as compared to female respondents (Fig. 3A–C). People who reported to be religious would agree to start a risky and uncomfortable treatment with a lower chance of survival for relatives (*p* = 0.034) as compared to those who are not religious, but religiosity did not influence any other answer (Fig. 3D). Having a nationality other than Austrian resulted in acceptance of a higher risk of severe disability for themselves (*p* = 0.014) and the acceptance of a lower chance of survival for patients to start a risky and uncomfortable treatment (*p* = 0.009) (Fig. 3E, F). Respondents with a university education (*p* < 0.0001 compared to apprenticeship, *p* = 0.016 compared to high school) or high school education (*p* = 0.008 compared to apprenticeship) would agree to start a risky and uncomfortable treatment for themselves with a lower chance of survival. A comparable result was obtained for the necessary survival chance of relatives (Fig. 3G, H).

### Influence of professional or personal exposure to critical care on responses regarding necessary chances of survival and acceptable risks of critical care treatments

**Fig. 4:**
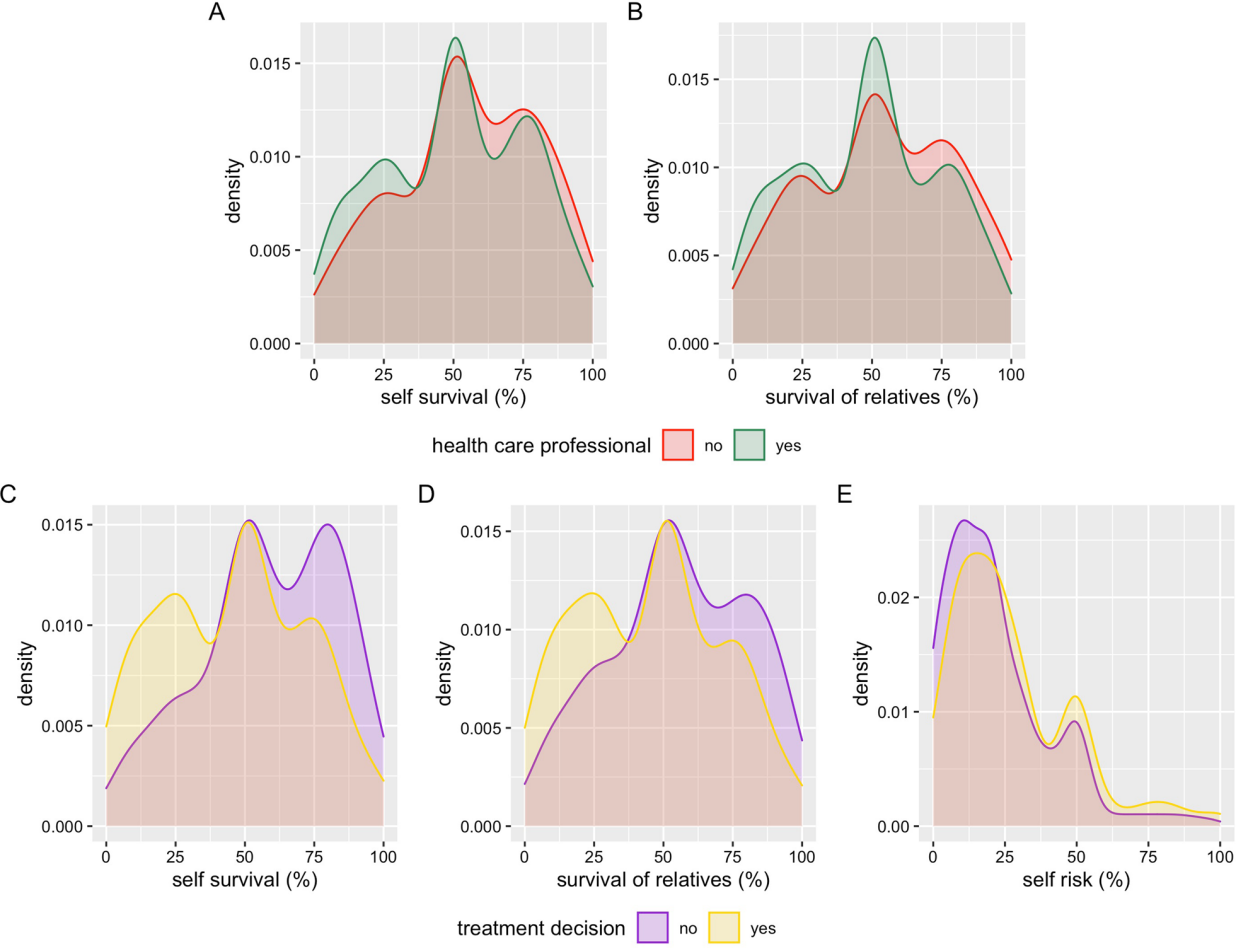
Density plots showing the distribution of responses to the different questions in relation to health care-related demographic variables (only significant differences are shown): **A**, **B** necessary survival for the respondents themselves and relatives to undergo a risky or uncomfortable therapy when critically ill in relation to being a health care professional or not; **C**–**E** necessary survival for the respondents themselves and relatives to undergo a risky or uncomfortable therapy as well as the acceptable risk for severe disability for themselves when critically ill in relation to being a being professionally involved in treatment decisions or not

Health care professionals would agree to start a risky and uncomfortable treatment with a lower chance of survival for themselves and for relatives (both *p* = 0.023) compared to non-health care professionals (Fig. 4A, B). When analysing the results separately for female and male respondents, female health care professionals did not respond differently to non-health care professionals, whereas male health care professionals would accept a lower chance of survival for themselves and for relatives compared to male non-medical respondents. Those who stated that they are professionally involved in treatment decisions of critically ill patients would accept to begin a risky and uncomfortable treatment with a lower chance of survival for themselves (*p* < 0.0001) and for relatives (*p* < 0.0001), and would accept a higher risk of severe disability (*p* = 0.002) (Fig. 4C–E). No sex-specific patterns were observed when comparing people who are professionally involved in treatment decisions with those who are not involved. Neither having experienced a critical illness personally nor with a close relative or friend influenced the answers on the necessary survival chance and the acceptable risk of disability. In addition, personal involvement in treatment decisions either when self-affected or for close relatives or friends did not influence the answers on the necessary chances of survival and risk of disability.

### Multivariate analysis of factors influencing responses regarding necessary chances of survival and acceptable risks of critical care treatments

The chances of survival necessary to accept a risky or uncomfortable procedure for the respondents themselves were influenced by sex (*p* < 0.001), education (*p* < 0.001) and making treatment decisions for patients (*p* < 0.001). In a multivariate model including sex, education and being a health care professional, only sex and education independently influenced the opinion of the participants (Table 3). Because sex, education and being a health care professional were not independent of each other, subgroup analysis was done to validate the results of the regression model. The acceptable chances of survival to accept a risky or uncomfortable procedure of university educated health care professionals involved in treatment decisions (*n* = 406) was only influenced by sex (*p* = 0.002) while sex did not play a role for university-educated participants who were not health care professionals (*p* = 0.212). For health care professionals who were not involved in treatment decisions (*n* = 220), the main explanatory variable was religiosity (*p* = 0.020). Participants who claimed not to be religious were more likely to choose medium or high necessary chances of survival to accept a risky or uncomfortable procedure compared to religious participants (OR = 2.167, 95%CI 1.001–4.728, *p* = 0.05 and OR = 2.919, 95%CI 1.355–6.289, *p* = 0.006, for medium and high necessary chances of survival, respectively). For health care professionals with a university-level education and involved in treatment decisions, the driving force was sex (*p* < 0.001); female participants were more likely to choose medium or high necessary chances of survival compared to male participants (OR = 2.522, 95%CI 1.598–3.980, *p* < 0.001 and OR = 1.752, 95%CI 1.046–2.934, *p* = 0.033, for medium and high chances of survival, respectively). For university-educated participants who were not health care professionals as well as for health care professionals who were not professionally involved in treatment decisions for critically ill patients, no prominent influencing factor could be identified. Further results of the multivariate analysis and subgroup analysis are given in the Additional file 2: Tables S1, S2 and S3.

**Table 3:** Multivariate multinomial logistic regression model for necessary survival chances to accept a risky and uncomfortable procedure during critical illness

Variable	Comparisons	Wald	Adjusted odds ratio	95% confidence interval		Adjusted *p* value
*Medium necessary survival chance*						
Constant		3.469				0.063
**Sex**	**Female compared to male**	**10.991**	**1.714**	**1.247**	**2.358**	**0.001**
**Education**	**Lower education compared to university education**	**8.622**	**1.728**	**1.199**	**2.489**	**0.003**
Health care professional	No compared to yes	0.492	1.145	0.784	1.671	0.483
*High necessary survival chances*						
Constant		15.817				< 0.001
**Sex**	**Female compared to male**	**17.167**	**2.024**	**1.450**	**2.826**	**< 0.001**
**Education**	**Lower education compared to university education**	**17.321**	**2.185**	**1.512**	**3.158**	**< 0.001**
Health care professional	No compared to yes	1.207	1.241	0.844	1.823	0.272

The lowest tercile (0–33%) of necessary survival chances was chosen as comparator. Independent predictors are printed in bold

The multinomial logistic regression model used to predict the acceptable disability risks to start a risky and uncomfortable treatment identified being a health care professional as the only significant influencing factor (*p* = 0.045). Participants who do not have a health care profession were more likely to accept a higher risk of disability to save their lives compared to health care professionals (OR = 1.869, 95%CI 1.036–3.370, 0.038, for medium and high risk, respectively). No other factor showed significant influence on this decision, neither in the whole study population nor when health care professionals and non-health care professionals were examined separately.

Because sex was such a prominent influencing factor on most of the categories, we further analysed female and male participants separately. For male participants, a university education (*p* = 0.002), being a health care professional (*p* = 0.006) and being professionally involved in treatment decisions for critically ill patients (*p* = 0.002) were the most influential factors for determining which chances of survival to accept a risky or uncomfortable treatment were necessary for themselves. For female participants, education (*p* = 0.001), being professionally involved in treatment decisions (*p* = 0.004) and religiosity (*p* = 0.003) were the most influential factors in this decision. In a multivariate model including education and religiosity, both proved to be independent influencing factors. In a multivariate model including religiosity and being professionally involved in treatment decisions, both factors also independently predict the acceptable necessary chances of survival. All relevant odds ratios are listed in Additional file 2: Table S4 and multivariate models in the subsequent Additional file 2: Tables S5 and S6.

### Relation between responses regarding necessary chances of survival and acceptable risks of critical care treatments

When comparing the answers regarding the acceptable chances of survival for the respondents, for relatives and for patients to start a risky and uncomfortable treatment, respondents would start a risky and uncomfortable treatment for patients with lower chances of survival than for themselves or relatives (*p* < 0.0001). The chosen acceptable risk of severe disability was higher for the respondents themselves compared to the patients (*p* < 0.0001). The necessary chance of survival for the respondents themselves, for relatives and for patients positively correlated with each other (*r* = 0.870, *p* < 0.0001 and *r* = 0.657, *p* < 0.0001, respectively). In addition, the acceptable risk of severe disability for oneself correlates with the result for patients (*r* = 0.452, *p* < 0.0001). A weak negative correlation between the necessary chances of survival for themselves and for relatives with the acceptable risk of disability was also observed (*r* =  − 0.127, *p* < 0.0001 and *r* =  − 0.128, *p* < 0.0001, respectively).

**Fig. 5:**
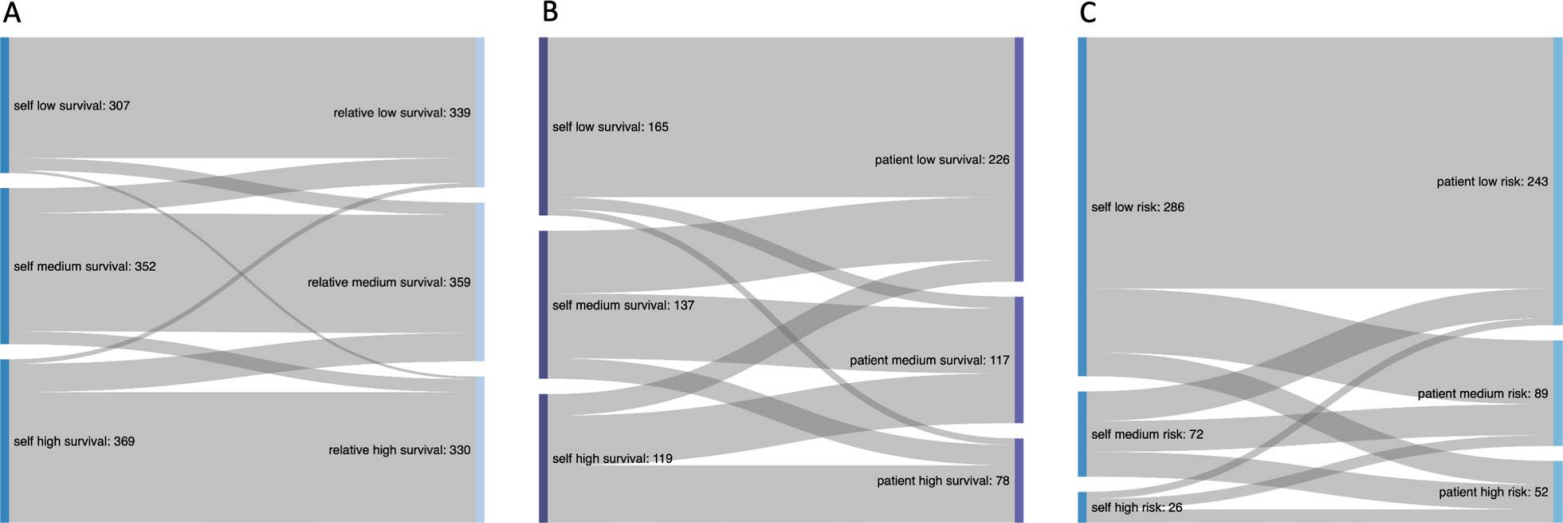
Sankey diagrams showing the flow between categories between different questions. The answers were grouped into low survival/risk (0–37.5%), medium survival/risk (37.6–62.5%) and high survival/risk (62.6–100%): **A** necessary survival to start a risky or uncomfortable treatment for the respondents themselves and for a close relative (*n* = 1,028 paired answers); **B** necessary survival to start a risky or uncomfortable treatment for the respondents themselves and for a patient (*n* = 421 paired answers); **C** acceptable risk for severe disability for the respondents themselves and for patients (*n* = 384 paired answers)

A substantial proportion of respondents would consider either lower (28%) or higher (18%) chances of survival necessary to start a risky or uncomfortable treatment for their relatives or their patients (21% lower and 10% higher). When asked for the acceptable risk of disability, 17% would accept a higher and 11% a lower risk of disability for patients than for themselves. (Fig. 5) Differing answers for the necessary chances of survival for respondents themselves compared to relatives were not influenced by any of the demographic variables. Differing answers for the necessary chances of survival of respondents themselves compared to patients was influenced by sex: 80% of female participants and 68% of male participants gave a differing answer for patients compared to themselves. No influence of demographic variables on the difference between the acceptable risk of disability for the respondents themselves compared to patients was observed.

## Discussion

Decision making in critical care is a complex task. While the goal of such decisions—performing treatments that have a reasonable expectation for survival outside the acute care setting with sufficient cognitive ability to perceive the benefits of treatment—are clearly set, it is difficult to quantify what a “reasonable” expectation for survival is. Previous studies aimed to quantify “futility” or “potential inappropriateness” of treatment, which can be considered as the opposite of “reasonable” expectation for survival. We aimed to identify, which chances of survival people consider necessary and which risk of disability is acceptable for them. To achieve this, we performed a survey that returned 1,052 responses from a random sample of health care professionals and people outside the medical field to gain insights into the quantitative aspects of “reasonable” survival and acceptable risk.

The concept of medical “futility” or “appropriateness of therapy” in critical care and intensive care medicine arises when it becomes clear that not everything that is technically possible is beneficial for patients [19]. Health care professionals have a well-formed and consistent qualitative opinion on the definition of futile care [20]. However, it is difficult to quantify “appropriateness of therapy”, which is the recommended term by a multidisciplinary statement from 2015 [8]. Furthermore, the concept of “inappropriateness of therapy” is critically discussed and challenged by differing views and expectations of health care professionals, patients and surrogates, and by limitations in prognostications [7, 19]. It has already been suggested by Schneiderman et al. in 1990 that a treatment should be considered “futile” when the chance for success is less than 1% [21]. While this is easily written down, it requires a very thorough knowledge of the outcome of a treatment in the individual patient on the one hand and may also be seen very differently by different individuals. The exact outcome of a treatment can usually not be predicted with reasonable certainty. Outcome predictions can vary considerably between predictive tools, physicians and nurses, with “human-made” prediction being superior to using predictive tools alone [22]. The “1% survival chance” to decide about inappropriateness of therapy of critical care has been challenged by a multicentre, mixed qualitative and quantitative study in the USA, where a substantial proportion (32%) of surrogates of critically ill patients elected to continue therapy when survival chances were less than 1% and 18% even elected for treatment to be continued when physicians believed that the patient had no chance to survive [16]. Our survey challenges the 1% survival chance cut-off for inappropriateness of therapy further as the range of answers span from 0 to 100% for each question asked, indicating a maximum of variation resulting in the impossibility to define a cut-off. Our study is unique in the sense that we did not provide preformed numerical answers but instead allowed the respondents to choose a number between 0 and 100% on a slide bar. Interestingly, a three-peak distribution pattern was observed for each question. The distribution of the peaks in the whole group of respondents shows an interesting pattern. While respondents for themselves and for their close relatives most commonly would start a risky or uncomfortable treatment when the chances of survival are between 34 and 66%, health care professionals would start a risky or uncomfortable treatment for their patients when the chances of survival are between 0 and 33%. This indicates that in general, respondents would want a higher chance of survival when making this decision for themselves or relatives than in the professional setting. However, for both themselves and for their patients, respondents would most commonly choose to start a risky or uncomfortable treatment only when there is a low (0–33%) risk of disability. While the relation seems “logical” for the decisions made by the respondents for themselves (i.e., the risk of disability should be low and treatments should only be initiated when survival chances are at least in a medium range), decisions in the professional setting for patients may cause inner conflicts for health care professionals: treatment should be started although chances of survival are only low but the acceptable risk of disability should also be low. Factors other than medical necessity, such as pressure from superiors or peers, families or threat of legal action have been identified in an Italian multicentre study as main non-medical reasons for inappropriate admissions to intensive care units [23].

In our study, answers were influenced by sex, religiosity and education as well as being a health care professional and being professionally involved in treatment decisions in critically ill, whereas having personal experience with critical illness only had a minor impact. Regarding sex, our results support the notion that for women, the chances for success of a therapy need to be higher and the potential risk needs to be lower than for men. This observed sex difference relates to several studies that observed sex-related differences in ICU admission rate as well as outcome. Male patients were more often admitted to ICU than female patients [24]; a female patient–female physician combination resulted in the lowest likelihood of being admitted to the ICU [25]. Sex also influences attitude toward end-of-life care [26]. Our study adds to this knowledge that sex is an important factor influencing our opinion on the appropriateness of critical care treatment.

Another noteworthy sex aspect of our study was that the opinion on necessary chances of survival to accept a risky procedure was influenced by sex in university-educated health care professionals involved in treatment decisions (who are most likely physicians working in critical care medicine), whereas sex did not play a role for university-educated participants who were not health care professionals. We can only speculate on the reasons for this interplay between sex and education as our study was not designed to elucidate any personality traits. Critical care medicine is a medical specialty where women remain underrepresented [27]. The motivation of students to choose medical schools has been studied extensively and reviewed [28]. Sex influences motivation independent of age, maturity and educational background [29]. Taken together, sex may influence the choice to study medicine and also the choice of medical specialty, and this may account for the differences we found between university-educated health care professionals and non-health care professionals in our study. It would be of interest to study differences in personality traits of female health care professionals compared to female non-health care professionals to explain these differences further.

Male health care professionals in our study would start a risky and uncomfortable treatment also in situations with a low survival chance. Having unrealistic views on the success of intensive care treatment may lead to an “escalation of commitment”—a business term that describes the continued investment of resources into a project even after there is objective evidence of the project’s impending failure. In critical care, the escalation of commitment may result in “doing everything possible” in a futile situation. Factors influencing escalation of commitment in business could be personality type, individual experience and sex [30]. Reasons for sex differences could lie in the decision making process itself, in the perception of appropriateness of treatment and also in general risk behaviour; however, data on personality traits and general risk behaviour have not been collected in our study.

When looking at the answers of men and women separately, religiosity influenced the answers regarding the necessary survival chances for themselves in female respondents only. Self-reported religiosity also had a slight but significant influence on the opinion on necessary survival of close relatives in the whole study population. In our study, self-reported religiosity was associated with lower necessary chances of survival to start a risky or uncomfortable treatment while a higher surrogate intrinsic religiosity was associated with lower patient receipt of life-sustaining treatments in adults and children in a study from the USA [31]. In a Canadian study in cancer patients, family caregivers and oncologists, male sex and having no religion was associated with approval of withholding life-sustaining measures [32]. In our multivariate subgroup analysis, religiosity was a significant explanatory variable in female but not in male respondents, indicating sex-specific differences in the importance of religiosity in such decisions. Interestingly, people who had experienced critical illness themselves or had close relatives who were critically ill considered themselves more often as religious in our study. Spirituality and spiritual care play an essential role in the treatment of critically ill patients and their families [33]. To the best of our knowledge, an association between previous experience of critical illness and religiosity has only been described in one qualitative study in a Muslim country so far [34].

University education, being a health care professional and being involved in treatment decisions are strongly interrelated variables in our study. By building different multinomial regression models we were able to show from our dataset that education strongly influences the answers in both male and female respondents. We hypothesize that this difference is most likely attributable to different opinions between nurses and physicians. Being female and working with critically ill patients without having university education corresponds most commonly to being a nurse in our survey as the majority of currently practicing nurses have been educated in nursing schools without obtaining a university degree in Austria. Inter-professional concordance on the provision of critical care perceived to be futile was low in a study where nurses and physicians were asked to judge independently [35, 36]. Patients categorised by nurses as receiving futile treatment had a lower 6-month mortality compared to those judged by physicians [35]. Different perceptions on inappropriateness of care between nurses and physicians are common [37]. Nurses feel better prepared for end-of-life decisions in critical care [38]. Taken together, our data support the notion of different viewpoints of different groups of health care professionals on treatment decisions in critical care and support the concept that especially in critical care, team discussions and shared decisions are necessary and beneficial for team members and patients [14] as part of a bioethical framework to guide the decision making process in critical care [2].

Interestingly, a proportion of respondents have differing opinions on necessary survival chances and acceptable risks for themselves, relatives and patients. It is well documented that for approximately one-third of ICU patients, there is disagreement between clinicians and patients/surrogates about the appropriateness of treatment. Disagreement about the appropriateness of treatment was associated with prognostic discordance and lower patient/surrogate satisfaction. Patients/surrogates who reported inappropriate treatment also reported lower satisfaction and trust in the medical team [36]. Our data show that disagreement may not only be found between different stakeholders in a multiprofessional team, but also one individual may have differing views on the necessary chances of survival and the acceptable risk for disability for themselves as compared to their relatives or patients. A similar “internal discordance” has been described for surgeons who were more likely to recommend surgery for a patient than they would choose surgery for themselves [39]. The impact of this internal discordance of opinions has so far not been studied yet but may be an interesting field for further study to understand the reasons as well as the impact on work satisfaction of health care professionals.

Our study has some limitations. As in all anonymous online surveys, we have to trust that people answer correctly regarding their demographics. We decided to use the whole dataset even when data seem to be unrealistic to us (e.g., there was a small group of people reporting to have compulsory education only and being involved in treatment decisions of critically ill patients, which we consider unlikely) because it was not possible to verify or falsify respondents´ answers and the exclusion of participants may introduce a bias. To keep the questionnaire short and, therefore, attractive for a large number of respondents, we only asked for broad categories of demographic data and did not include any detailed assessment of different dimensions (e.g., for religiosity and religion) or psychometric assessments. Although we aimed to recruit a diverse group of participants, the majority of respondents came from Austria; therefore, the results may not be generalizable for other countries or cultures. Finally, there may be a selection bias in the respondents because the questionnaire was distributed anonymously to a random sample and we have no data on people who received but did not submit the survey, which is again a limitation that our questionnaire shares with other anonymous surveys. Because the questionnaire was distributed by a female (VS) and a male (MZ) researcher, we believe that we introduced no sex-specific selection bias.

## Conclusion

Our study shows that health care professionals as well as non-medical people have very divergent views on the chances of survival they consider necessary to start a risky and uncomfortable treatment as well as on the acceptable risks for such a treatment for themselves, close relatives and patients. Sex, education, being a health care professional, being involved in treatment decisions and religiosity influence these opinions. No cut-off can be identified for the chances of survival or acceptable risk of disability to start a risky or uncomfortable treatment because all answers covered the whole range between 0 and 100%. The results of our study may assist the decision-making process related to appropriateness of therapy in a way that the distribution of answers shows no upfront “right” or “wrong” decision in relation to the necessary chances of survival to start intensive care treatment. Although it may sound like a platitude, communication within multiprofessional teams, with patients and with relatives in each individual case, seems to be the key to understand the viewpoints of all stakeholders in such a process. Hospitals or even legal policies for a decision making process are of value in such a discussion process, but need to consider more than “just” improving clinical decision making by creating better or easier prognosis models in critical care. Health care professionals need to be trained to adequately assess values and incorporate the opinion of patients or surrogates into such a process, and to interpret their opinions in respect to their personal and professional background. While our data give a first indication which factors influence the opinion on the necessary chances of survival and the acceptable risk to start intensive care treatment, further quantitative and qualitative studies are needed for a deeper understanding of influencing factors such as personality traits and the professional role.

## Supplementary Information

**Additional file 1.** The questionnaires in German and English.

**Additional file 2.** The supplementary data.

## Data Availability

The datasets used and/or analysed during the current study are available from the corresponding author on reasonable request.
