# Supplementary material for: When is it considered reasonable to start a risky and uncomfortable treatment in critically ill patients? A random sample online questionnaire study
Source: BMC Med Ethics. 2021 Nov 3;22:146. doi: 10.1186/s12910-021-00705-4 (PMC8564596; doi:10.1186/s12910-021-00705-4)
Supplement: Supplementary file 1 — Additional file 1. The questionnaires in German and English. [file 12910_2021_705_MOESM1_ESM.docx]

# Online survey on therapeutic goals in critically ill

## Online survey on therapeutic goals in critically ill patients

## (untitled)

### 1) How old are you?

_________________________________________________

#### 2) What is your gender?

( ) male

( ) female

#### 3) Which religion do you belong to?

( ) Christian

( ) Islam

( ) Jewish

( ) Hindu

( ) Buddhism

( ) Other - Write In: _________________________________________________

#### 4) Do you consider yourself to be religious?

( ) Yes

( ) No

#### 5) Which country are you from?

( ) Afghanistan

( ) Albania

( ) Algeria

( ) Andorra

( ) Angola

( ) Antigua and Barbuda

( ) Argentina

( ) Armenia

( ) Australia

( ) Austria

( ) Azerbaijan

( ) Bahamas, The

( ) Bahrain

( ) Bangladesh

( ) Barbados

( ) Belarus

( ) Belgium

( ) Belize

( ) Benin

( ) Bhutan

( ) Bolivia

( ) Bosnia and Herzegovina

( ) Botswana

( ) Brazil

( ) Brunei

( ) Bulgaria

( ) Burkina Faso

( ) Burundi

( ) Cambodia

( ) Cameroon

( ) Canada

( ) Cape Verde

( ) Central African Republic

( ) Chad

( ) Chile

( ) China

( ) Colombia

( ) Comoros

( ) Congo, Democratic Republic of the

( ) Congo, Republic of the

( ) Costa Rica

( ) Cote d'Ivoire

( ) Croatia

( ) Cuba

( ) Curacao

( ) Cyprus

( ) Czech Republic

( ) Denmark

( ) Djibouti

( ) Dominica

( ) Dominican Republic

( ) East Timor (see Timor-Leste)

( ) Ecuador

( ) Egypt

( ) El Salvador

( ) Equatorial Guinea

( ) Eritrea

( ) Estonia

( ) Ethiopia

( ) Fiji

( ) Finland

( ) France

( ) Gabon

( ) Gambia, The

( ) Georgia

( ) Germany

( ) Ghana

( ) Greece

( ) Grenada

( ) Guatemala

( ) Guinea

( ) Guinea-Bissau

( ) Guyana

( ) Haiti

( ) Holy See

( ) Honduras

( ) Hong Kong

( ) Hungary

( ) Iceland

( ) India

( ) Indonesia

( ) Iran

( ) Iraq

( ) Ireland

( ) Israel

( ) Italy

( ) Jamaica

( ) Japan

( ) Jordan

( ) Kazakhstan

( ) Kenya

( ) Kiribati

( ) Kosovo

( ) Kuwait

( ) Kyrgyzstan

( ) Laos

( ) Latvia

( ) Lebanon

( ) Lesotho

( ) Liberia

( ) Libya

( ) Liechtenstein

( ) Lithuania

( ) Luxembourg

( ) Macau

( ) Macedonia

( ) Madagascar

( ) Malawi

( ) Malaysia

( ) Maldives

( ) Mali

( ) Malta

( ) Marshall Islands

( ) Mauritania

( ) Mauritius

( ) Mexico

( ) Micronesia

( ) Moldova

( ) Monaco

( ) Mongolia

( ) Montenegro

( ) Morocco

( ) Mozambique

( ) Myanmar

( ) Namibia

( ) Nauru

( ) Nepal

( ) Netherlands

( ) Netherlands Antilles

( ) New Zealand

( ) Nicaragua

( ) Niger

( ) Nigeria

( ) North Korea

( ) Norway

( ) Oman

( ) Pakistan

( ) Palau

( ) Palestinian Territories

( ) Panama

( ) Papua New Guinea

( ) Paraguay

( ) Peru

( ) Philippines

( ) Poland

( ) Portugal

( ) Qatar

( ) Romania

( ) Russia

( ) Rwanda

( ) Saint Kitts and Nevis

( ) Saint Lucia

( ) Saint Vincent and the Grenadines

( ) Samoa

( ) San Marino

( ) Sao Tome and Principe

( ) Saudi Arabia

( ) Senegal

( ) Serbia

( ) Seychelles

( ) Sierra Leone

( ) Singapore

( ) Slovakia

( ) Slovenia

( ) Solomon Islands

( ) Somalia

( ) South Africa

( ) South Korea

( ) South Sudan

( ) Spain

( ) Sri Lanka

( ) Sudan

( ) Suriname

( ) Swaziland

( ) Sweden

( ) Switzerland

( ) Syria

( ) Taiwan

( ) Tajikistan

( ) Tanzania

( ) Thailand

( ) Timor-Leste

( ) Togo

( ) Tonga

( ) Trinidad and Tobago

( ) Tunisia

( ) Turkey

( ) Turkmenistan

( ) Tuvalu

( ) Uganda

( ) Ukraine

( ) United Arab Emirates

( ) United Kingdom

( ) United States

( ) Uruguay

( ) Uzbekistan

( ) Vanuatu

( ) Venezuela

( ) Vietnam

( ) Yemen

( ) Zambia

( ) Zimbabwe

#### 6) What is your highest level of education?

( ) Compulsory education

( ) Apprenticeship

( ) High school

( ) University

#### 7) Are you a health care worker?

( ) Yes

( ) No

#### 8) Do you work with critically ill patients?

( ) Yes

( ) No

#### 9) Are you involved in treatment decisions for critically ill patients?

( ) Yes

( ) No

#### 10) Have you ever been critically ill or in a life-threatening situation yourself?

( ) Yes

( ) No

#### 11) Were you involved in treatment decisions in this situation?

( ) Yes

( ) No

#### 12) Has a person closely related to you ever been critically ill or in a life-threatening situation?

( ) Yes

( ) No

#### 13) Were you as a related person involved in treatment decisions in this situation?

( ) Yes

( ) No

## (untitled)

#### 14) Imagine that you are in hospital with a life-threatening disease. For which survival chance would you undergo a risky and uncomfortable treatment?

0 ________________________[__]_____________________________ 100

#### 15) Imagine that you have to decide for a close friend or relative who is in hospital with  a life-threatening disease. For which survival chance would you decide that this person should undergo a risky and uncomfortable treatment?

0 ________________________[__]_____________________________ 100

#### 16) Imagine you are in a situation for which a risky and uncomfortable treatment will ensure survival, but there is a risk of severe disability after the treatment.  Which risk of severe disability with the need for long-term care would you consider acceptable?

0 ________________________[__]_____________________________ 100

## (untitled)

#### 17) Imagine you are treating a critically ill patient who is unconscious.   Which survival chance is necessary for you to begin a risky and uncomfortable treatment on this patient?

0 ________________________[__]_____________________________ 100

#### 18) Imagine you are treating a critically ill patient who is unconscious and for whom treatment may result in severe disability.   Which risk of severe disability needing long-term care is acceptable?

0 ________________________[__]_____________________________ 100

## Thank you!
