# Supplementary material for: When is it considered reasonable to start a risky and uncomfortable treatment in critically ill patients? A random sample online questionnaire study
Source: BMC Med Ethics. 2021 Nov 3;22:146. doi: 10.1186/s12910-021-00705-4 (PMC8564596; doi:10.1186/s12910-021-00705-4)
Supplement: Supplementary file 2 — Additional file 2. The supplementary data. [file 12910_2021_705_MOESM2_ESM.docx]

**Supplementary data**

**When is it considered reasonable to start a risky and uncomfortable treatment in critically ill patients? A random sample online questionnaire study**

M. Zink^1*^, A. Horvath^2,3*^, V. Stadlbauer^2,3^

1. Department of Anaesthesiology and Intensive Care Medicine, Hospital of the Brothers of St. John of God, St.Veit an der Glan, Austria and Hospital of the Elisabethinen Klagenfurt, Klagenfurt, Austria
2. Department of Internal Medicine, Research Unit „Transplantation Research“, Medical University of Graz
3. Center for Biomarker Research in Medicine (CBmed), Graz, Austria

* Contributed equally to the paper

Supplementary methods

Email invitations to participate in the survey were sent to the following institutions, clubs and communities.

Club 41 Austria, Hospitals of the Brothers of St. John of God Austria, Transplantforum Upper Austria. ÖGARI (Austrian Society of Anesthesiology, Rescusitation and Intensive Care, University Mainz, Germany, Zuoz Club, Switzerland, Alevitic Community Austria, Old Catholic Church Austria, Armenian-apostolic community Austria, Baha’i Community Austria, Christian Community of Austria, Unification Church in Austria, Evangelical Church in Austria, Methodist Church in Austria, Free churches in Austria, Israelitische Kultusgemeinde Vienna, Islamic Faith Community Austria, Islamic-Shiite religious community in Austria, Church of Jesus Christ Austria, New Apostolic Church Austria, Orthodox Church Austria, Austrian Bishops' Conference, Austrian Buddhist religious community, Pentecostal Church Austria, Romanian Orthodox Church Austria, Russian Orthodox Church Austria, Syrian Orthodox Church Austria, Jehovah's Witnesses Austria

**Multivariate analysis of factors influencing the answers regarding necessary survival chances and acceptable risks of critical care treatments**

In addition to the model we described in the main text, only health care professionals were analysed to test the influence of being involved in treatment decision and sex on the opinion on the necessary chances of survival of the respondents for themselves to start a risky or uncomfortable treatment. Education was excluded from the model because of its strong associations with treatment decision experience. Both, sex (p=0.001) and being professionally involved in treatment decisions (p<0.001) were independently influencing the opinion on the necessary chances of survival. (supplementary Table 1) Similar results were observed when the participants were asked about the necessary chances of survival to start a risky or uncomfortable treatment in a relative. (supplementary table 2).

Supplementary Table 1: Multivariate multinomial logistic regression model for necessary chances of survival to accept a risky and uncomfortable procedure during critical illness for themselves in people with health care professions. The lowest tercile of necessary survival chances was chosen as comparator. Independent predictors are printed in bold.

| Variable | Comparisons | Wald | adjusted  odds ratio | 95% confidence interval | | adjusted p-value |
| --- | --- | --- | --- | --- | --- | --- |
| **Medium necessary survival chances** | | | | | | |
| Constant |  | 7.935 |  |  |  | 0.005 |
| **Sex** | **female compared to male** | **11.973** | **2.030** | **1.359** | **3.031** | **0.001** |
| **Professionally involved in treatment decisions** | **no compared to yes** | **7.850** | **1.906** | **1.214** | **2.992** | **0.005** |
| **High necessary survival chances** | | | | | | |
| Constant |  | 16.498 |  |  |  | <0.001 |
| **Sex** | **female compared to male** | **7.725** | **1.817** | **1.193** | **2.769** | **0.005** |
| **Professionally involved in treatment decisions** | **no compared to yes** | **20.620** | **2.881** | **1.825** | **4.548** | **<0.001** |

Supplementary table 2: Multivariate multinomial logistic regression model for necessary chances of survival to accept a risky and uncomfortable treatment for a relative in health care professionals. The lowest tercile of necessary survival chances was chosen as comparator. Independent predictors are printed in bold.

| Variable | Comparisons | Wald | adjusted  odds ratio | 95% confidence interval | | adjusted p-value |
| --- | --- | --- | --- | --- | --- | --- |
| **Medium necessary survival chances** | | | | | | |
| Constant |  | 9.318 |  |  |  | 0.002 |
| **Sex** | **female compared to male** | **12.928** | **2.052** | **1.387** | **3.037** | **<0.001** |
| **Professionally involved in treatment decisions** | **no compared to yes** | **5.829** | **1.690** | **1.104** | **2.588** | **0.016** |
| **High necessary survival chances** | | | | | | |
| Constant |  | 21.048 |  |  |  | <0.001 |
| **Sex** | **female compared to male** | **6.255** | **1.720** | **1.124** | **2.631** | **0.012** |
| **Professionally involved in treatment decisions** | **no compared to yes** | **12.713** | **2.264** | **1.445** | **3.547** | **<0.001** |

When health care professionals were asked which chances of survival they consider necessary for patients to start a risky or uncomfortable treatment, the answers were mainly influence by sex (p=0.007) and education (p=0.020). Female participants were more likely to require medium chances of survival compared to male participants and non-university educated participants were more likely to require high necessary chances of survival to university educated participants to agree to start a risky or uncomfortable treatment. This pattern was validated in a multivariate model (supplementary table 3)

Table 3: Multivariate multinomial logistic regression model for necessary chances of survival to accept a risky and uncomfortable procedure for a patient. The lowest tercile of necessary survival chances was chosen as comparator. Independent predictors are printed in bold.

| Variable | Comparisons | Wald | adjusted  odds ratio | 95% confidence interval | | adjusted p-value |
| --- | --- | --- | --- | --- | --- | --- |
| **Medium necessary survival chances** | | | | | | |
| Constant |  | 29.232 |  |  |  | <0.001 |
| **Sex** | **female compared to male** | **7.482** | **1.881** | **1.196** | **2.958** | **0.006** |
| Education | non-university compared to university education | 2.652 | 2.560 | 0.826 | 7.934 | 0.103 |
| **High necessary survival chances** | | | | | | |
| Constant |  | 45.112 |  |  |  | <0.001 |
| Sex | female compared to male | 1.222 | 1.363 | 0.787 | 2.359 | 0.269 |
| **Education** | **non-university compared to university education** | **6.583** | **4.638** | **1.437** | **14.976** | **0.010** |

Supplementary table 4: Odds ratios of relevant factors in the decision of necessary chances of survival to accept a risky and uncomfortable treatment for themselves; for female and male participants. The lowest tercile of necessary survival chances was chosen as comparator. Significant results are printed in bold.

|  | Female participants | | | | Male participants | | | |
| --- | --- | --- | --- | --- | --- | --- | --- | --- |
|  | medium necessary survival chances | | high necessary survival chances | | medium necessary survival chances | | high survival necessary chances | |
| Variable | OR (95%CI) | p-value | OR (95%CI) | p-value | OR (95%CI) | p-value | OR (95%CI) | p-value |
| education | **1.5 (1.0-2.4)** | **0.049** | **2.2 (1.5-3.4)** | **<0.001** | **2.5 (1.4-4.6)** | **0.003** | **2.7 (1.4-5.0)** | **0.002** |
| health care profession | 0.9 (0.5-1.4) | 0.523 | 1.2 (0.7-1.9) | 0.461 | **2.3 (1.3-4.1)** | **0.003** | **2.0 (1.1-3.5)** | **0.025** |
| treatment decisions | 1.6 (0.9-2.6) | 0.100 | **2.4 (1.4-4.2)** | **0.001** | **3.3 (1.3-8.2)** | **0.010** | **4.3 (1.7-10.8)** | **0.002** |
| religiosity | **1.9 (1.3-2.9)** | **0.002** | **1.9 (1.2-2.9)** | **0.003** | 0.9 (0.6-1.4) | 0.631 | 1.0 (0.6-1.6) | 0.936 |

Supplementary table 5: Multivariate multinomial logistic regression model for necessary chances of survival for themselves in male participants. The lowest tercile of necessary survival chances was chosen as comparator. Independent predictors are printed in bold.

| Variable | Comparisons | Wald | adjusted  odds ratio | 95% confidence interval | | adjusted p-value |
| --- | --- | --- | --- | --- | --- | --- |
| **Medium survival chances** | | | | | | |
| Constant |  | 7.421 |  |  |  | 0.006 |
| **Education** | **lower education compared to university education** | **4.351** | **1.979** | **1.042** | **3.759** | **0.037** |
| Health care professional | No compared to yes | 4.439 | 1.875 | 1.045 | 3.366 | 0.035 |
| **High survival chances** | | | | | | |
| Constant |  | 14.556 |  |  |  | <0.001 |
| **Education** | **lower education compared to university education** | **5.306** | **2.199** | **1.125** | **4.300** | **0.021** |
| Health care professional | No compared to yes | 1.813 | 1.540 | 0.822 | 2.886 | 0.178 |

Supplementary table 6: Multivariate multinomial logistic regression model for necessary chances of survival for themselves in female participants. The lowest tercile of necessary survival chances was chosen as comparator. Independent predictors are printed in bold.

| Variable | Comparisons | Wald | adjusted  odds ratio | 95% confidence interval | | adjusted p-value |
| --- | --- | --- | --- | --- | --- | --- |
| **Medium survival chances** | | | | | | |
| Constant |  | 0.088 |  |  |  | 0.767 |
| **Education** | **lower education compared to university education** | **4.877** | **1.646** | **1.058** | **2.561** | **0.027** |
| **Religiosity** | **non-religious compared to religious** | **9.921** | **1.977** | **1.294** | **3.021** | **0.002** |
| **High survival chances** | | | | | | |
| Constant |  | 0.787 |  |  |  | 0.375 |
| **Education** | **lower education compared to university education** | **15.052** | **2.392** | **1.540** | **3.717** | **<0.001** |
| **Religiosity** | **non-religious compared to religious** | **10.167** | **2.011** | **1.309** | **3.090** | **0.001** |
